# The Gut Microbiota Communities of Wild Arboreal and Ground-Feeding Tropical Primates Are Affected Differently by Habitat Disturbance

**DOI:** 10.1128/mSystems.00061-20

**Published:** 2020-05-26

**Authors:** Claudia Barelli, Davide Albanese, Rebecca M. Stumpf, Abigail Asangba, Claudio Donati, Francesco Rovero, Heidi C. Hauffe

**Affiliations:** a Department of Biodiversity and Molecular Ecology, Research and Innovation Centre, Fondazione Edmund Mach, San Michele all’Adige, Italy; b Carl R. Woese Institute for Genomic Biology, University of Illinois, Urbana, Illinois, USA; c Tropical Biodiversity Section, MUSE – Museo delle Scienze, Trento, Italy; d Computational Biology Research Unit, Research and Innovation Centre, Fondazione Edmund Mach, San Michele all’Adige, Italy; e Department of Anthropology, University of Illinois, Urbana, Illinois, USA; f Department of Biology, University of Florence, Florence, Italy; Oregon State University

**Keywords:** Tanzania, Udzungwa, bacteria, conservation, fungi, gut microbiota, habitat degradation, primates, red colobus, yellow baboon

## Abstract

Human exploitation and destruction of tropical resources are currently threatening innumerable wild animal species, altering natural ecosystems and thus, food resources, with profound effects on gut microbiota. Given their conservation status and the importance to tropical ecosystems, wild nonhuman primates make excellent models to investigate the effect of human disturbance on the diversity of host-associated microbiota. Previous investigations have revealed a loss of fecal bacterial diversity in primates living in degraded compared to intact forests. However, these data are available for a limited number of species, and very limited information is available on the fungal taxa hosted by the gut. Here, we estimated the diversity and composition of gut bacterial and fungal communities in two primates living sympatrically in both human-modified and pristine forests in the Udzungwa Mountains of Tanzania. Noninvasively collected fecal samples of 12 groups of the Udzungwa red colobus (Procolobus gordonorum) (*n* = 89), a native and endangered primate (arboreal and predominantly leaf-eating), and five groups of the yellow baboon (Papio cynocephalus) (*n* = 69), a common species of least concern (ground-feeding and omnivorous), were analyzed by the V1-V3 region of the 16S rRNA gene (bacterial) and ITS1-ITS2 (fungal) sequencing. Gut bacterial diversities were associated with habitat in both species, most likely depending on their ecological niches and associated digestive physiology, dietary strategies, and locomotor behavior. In addition, fungal communities also show distinctive traits across hosts and habitat type, highlighting the importance of investigating this relatively unexplored gut component.

**IMPORTANCE** Gut microbiota diversity has become the subject of extensive research in human and nonhuman animals, linking diversity and composition to gut function and host health. Because wild primates are good indicators of tropical ecosystem health, we developed the idea that they are a suitable model to observe the consequences of advancing global change (e.g., habitat degradation) on gut microbiota. So far, most of the studies focus mainly on gut bacteria; however, they are not the only component of the gut: fungi also serve essential functions in gut homeostasis. Here, for the first time, we explore and measure diversity and composition of both bacterial and fungal microbiota components of two tropical primate species living in highly different habitat types (intact versus degraded forests). Results on their microbiota diversity and composition are discussed in light of conservation issues and potential applications.

## Abstract (precis)

Gut microbiota diversity has become the subject of extensive research in human and nonhuman animals, linking diversity and composition to gut function and host health. Because wild primates are good indicators of tropical ecosystem health, we developed the idea that they are a suitable model to observe the consequences of advancing global change (e.g., habitat degradation) on gut microbiota. So far, most of the studies focus mainly on gut bacteria; however, they are not the only component of the gut: fungi also serve essential functions in gut homeostasis. Here, for the first time, we explore and measure diversity and composition of both bacterial and fungal microbiota components of two tropical primate species living in highly different habitat types (intact versus degraded forests). Results on their microbiota diversity and composition are discussed in light of conservation issues and potential applications.

## INTRODUCTION

Human exploitation and destruction of natural ecosystems are causing the extinction of wild animal species on a global scale (1–3). Recent research also suggests that disturbing the habitat of wild animal populations may trigger the loss of “microbiodiversity,” the array of microorganisms hosted by various body niches, such as the skin and gut (4–6). Given that this microbiota, especially the bacterial component, is known to play a crucial role in animal development, immunity, and nutrition (7–9), loss of this microbiodiversity could have an effect on an individual’s behavior, health, and ultimately on its lifetime fitness (10, 11). In vulnerable animal species, a decrease in individual health and reproductive output could accelerate population decline and species extinction (12, 13).

Tropical nonhuman primates have been a particular focus of microbiota research, given their phylogenetic affinity to humans (14), but also for their contribution to forest regeneration and ecosystem health (15) and conservation status (11), with half of the species being classified as endangered or critically endangered (16, 17). In primates, gut bacterial communities are known to be species specific (18), but they vary widely with age (19), social structure (20), and diet (21, 22) with significant losses of bacterial diversity in captivity (23, 24). Gut microbiota composition and diversity have also been found to vary between contrasting habitat type (4, 5, 19, 25, 26). For example, populations living in fragments of both the black howler monkey (Alouatta pigra) (5) and Udzungwa red colobus (Procolobus gordonorum) (4) have been shown to have a lower gut bacterial diversity in fragmented habitats compared to intact habitats (but see reference 27 for Ugandan red colobus [Procolobus rufomitratus] and black and white colobus [Colobus guereza] and reference 26 for *Chlorocebus* monkeys). In red colobus, bacterial diversity loss is potentially linked to a decreased ability to digest toxic plant compounds (4), while the gut microbiota of black howler monkeys from secondary forests were more enriched by potentially pathogenic bacterial families, associated with malnutrition and disease (25). Similarly, ring-tailed lemurs (Lemur catta) living in marginal areas were found to be overrepresented by *Coprococcus* probably as a result of increased contact with human and domestic animal waste (19).

Until now, microbiota research has focused on the bacterial component of the gut ecosystem. Fungi are another relevant, yet relatively neglected taxa contributing to host immunity and gut health, and are possibly associated with disease susceptibility (for a review, see reference 28). In humans, specific fungal communities (“mycobiota”) have also been associated with obesity (29). The mycobiota in wild vertebrate populations are less explored (30, 31), but the composition and diversity of plant-degrading fungi in the gut appear to be linked with phylogeny in herbivorous mammals (32–38). Gut fungi have also recently been shown to vary with sex, age, and season in a group of macaques (39). However, the effect of habitat fragmentation and host lifestyle on gut fungi is unknown, as are the interactions between fungal and bacterial microbiota components.

The present research uses wild primates to investigate the impact of human disturbance and habitat fragmentation on bacterial and fungal gut communities. In this study, we characterize and compare the bacterial and fungal components of the gut microbiota in two nonhuman primate species, one arboreal (the Udzungwa red colobus [*Procolobus gordonorum*]), and one ground-feeding (the yellow baboon [*Papio cynocephalus*]) living in both protected and fragmented habitats using metataxonomics of noninvasive fecal samples.

Habitat quality is expected to be crucial in determining the diet ingested by the host, which in turn is one of the most plausible drivers of gut microbiota composition and diversity (4, 40–42); animals feeding in disturbed and fragmented forest tend to have less diverse food sources than in more pristine and continuous forests (43); forests surrounded by cultivations and livestock are also more likely affected by treatments of antimycotics, antibiotics, and pesticides. Thus, we tested whether aspects of primate ecology (diet and behavior) were associated with gut microbiota diversities, asking specifically the three following questions. (i) Are gut microbiota bacterial and fungal diversities higher in a leaf-eating, arboreal (the red colobus) host compared to an omnivorous, terrestrial (yellow baboon) host, as predicted (44, 45)? (ii) Are gut microbiota bacterial and fungal diversities higher in hosts inhabiting protected forests than in those living in fragmented and degraded ones? (iii) Can microbiota composition be used to predict sample origin?

## RESULTS

A total of 2,112,896 high-quality reads were obtained from 69 yellow baboon and 89 red colobus fecal samples. Among these reads, 973,226 reads (mean ± standard deviation [SD], 6,160 ± 2,042 reads/sample) belonged to bacteria, while 1,139,670 reads (7,213 ± 4,564 reads/sample) were fungal sequences. After rarefaction and classification, a total of 4,620 bacterial sequence variants (SVs) were recruited from all 158 samples, and 1,419 fungal SVs were recovered from 142 samples.

### Are gut microbiota bacterial and fungal diversities higher in a leaf-eating arboreal host (the red colobus) compared to an omnivorous, terrestrial (yellow baboon) host? (i) Bacteria: taxonomy, abundance, and diversity across hosts.

**FIG 1:**
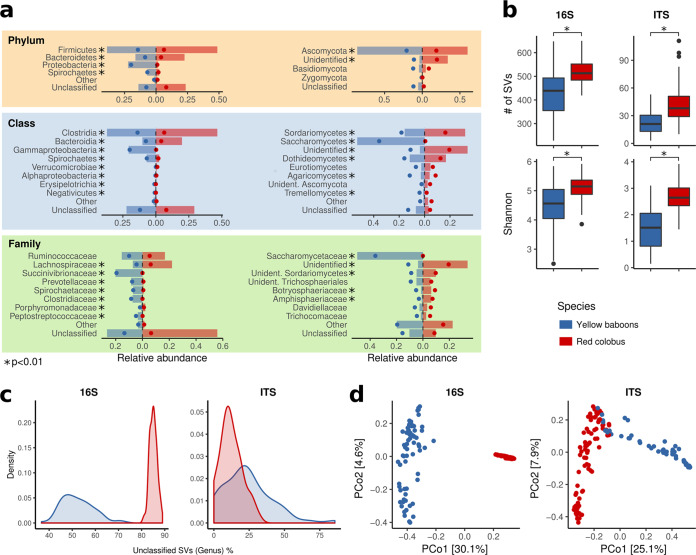
Taxonomic analyses of fecal bacteria (16S) and fungi (ITS) for red colobus (red in all four panels) and yellow baboon (blue). (a) Dominant phyla, classes, and families. The bars show the mean relative abundances expressed as percentages, while the dots show standard deviations. Unidentified refers to SVs present in the reference database; thus, SVs classified as kingdom Fungi for which no lower classification was possible. (b) Bacterial and fungal diversity indices. The number of observed SVs (richness) and Shannon entropy are depicted. (c) Distribution of percentage unclassified SVs at the genus level. (d) Principal coordinate analysis (PCoA) of Bray-Curtis dissimilarities. *, *P* < 0.01.

Of the 11 bacterial phyla detected, seven were identified in both primate species (*Actinobacteria*, *Bacteroidetes*, *Cyanobacteria*/chloroplast, *Firmicutes*, *Verrucomicrobia*, *Spirochaetes*, and *Proteobacteria*), while *Fusobacteria*, *Elusimicrobia*, *Tenericutes*, *Lentisphaerae*, and *Fibrobacteres* were found only in yellow baboons (Fig. 1a; see also Data Set S1 in the supplemental material [sheet 1] [no red colobus phyla were unique]).

10.1128/mSystems.00061-20.8 DATA SET S1 (Sheet 1) Median, first quartile (Q1), third quartile (Q3), mean and standard deviation (SD) of relative abundances of bacterial (16S) SVs at the phylum level in yellow baboons (*Papio cynocephalus*) and red colobus (*Procolobus gordonorum*). (Sheet 2) Median, first quartile (Q1), third quartile (Q3), mean and standard deviation (SD) of relative abundances of bacterial (16S) SVs at the class level in yellow baboons (*Papio cynocephalus*) and red colobus (*Procolobus gordonorum*). (Sheet 3) Wilcoxon rank sum test between yellow baboons (*Papio cynocephalus*) and red colobus (*Procolobus gordonorum*) of the relative abundances of 16S SVs at the phylum, class, and family levels. For each taxon, we report the median (“estimate” column), the 95% nonparametric confidence interval (“conf.low” and “conf.high” columns) of the relative abundances difference between the two species, the test statistic, the *P* value, and the FDR-corrected *P* value (“p.value.adj” column). (Sheet 4) Median, first quartile (Q1), third quartile (Q3), mean and standard deviation (SD) of relative abundances of bacterial (16S) SVs at the family level in yellow baboons (*Papio cynocephalus*) and red colobus (*Procolobus gordonorum*). (Sheet 5) Median, first quartile (Q1), third quartile (Q3), mean and standard deviation (SD) of relative abundances of fungal (ITS) SVs at the family level in yellow baboons (*Papio cynocephalus*) and red colobus (*Procolobus gordonorum*). (Sheet 6) Median, first quartile (Q1), third quartile (Q3), mean and standard deviation (SD) of relative abundances of fungal (ITS) SVs at the phylum level in yellow baboons (*Papio cynocephalus*) and red colobus (*Procolobus gordonorum*). (Sheet 7) Median, first quartile (Q1), third quartile (Q3), mean and standard deviation (SD) of relative abundances of fungal (ITS) SVs at the class level in yellow baboons (*Papio cynocephalus*) and red colobus (*Procolobus gordonorum*). (Sheet 8) Wilcoxon rank sum test between yellow baboons (*Papio cynocephalus*) and red colobus (*Procolobus gordonorum*) of the relative abundances of ITS SVs at the phylum, class, and family levels. For each SV, we report the median (“estimate” column), the 95% nonparametric confidence interval (“conf.low” and “conf.high” columns) of the difference in relative abundances between the two species, the test statistic, the *P* value, and the FDR-corrected *P* value (“p.value.adj” column). (Sheet 9) Median, first quartile (Q1), third quartile (Q3), mean and standard deviation (SD) of relative abundances of unclassified 16S and ITS SVs at the genus level. (Sheet 10) CCREPE results for yellow baboon (*Papio cynocephalus*). Significant correlations between 16S and ITS SVs calculated on each forest (Mwanihana, a PF, and Magombera, a FF). (Sheet 11) CCREPE results for red colobus (*Procolobus gordonorum*). Significant correlation between 16S and ITS SVs calculated on each forest (Mwanihana, a PF, and Magombera, a FF). (Sheet 12) MICTools results (yellow baboons). TIC_e_ (total information coefficient) *P* values (TICePVal), Pearson correlation coefficients (PearsonR), Spearman’s rank correlation coefficients (SpearmanRho), and maximal information coefficient (MICe) for each significant relationship between 16S SVs across forests. (Sheet 13) MICTools results (red colobus). TIC_e_ *P* values (TICePVal), Pearson correlation coefficients (PearsonR), Spearman’s rank correlation coefficients (SpearmanRho), and maximal information coefficient (MICe) for each significant relationship between 16S SVs across forests. (Sheet 14) MICTools results (yellow baboons). TIC_e_ *P* values (TICePVal), Pearson correlation coefficients (PearsonR), Spearman’s rank correlation coefficients (SpearmanRho), and maximal information coefficient (MICe) for each significant relationship between ITS SVs across forests. (Sheet 15) MICTools results (red colobus). TIC_e_ *P* values (TICePVal), Pearson correlation coefficients (PearsonR), Spearman’s rank correlation coefficients (SpearmanRho), and maximal information coefficient (MICe) for each significant relationship between ITS SVs across forests. (Sheet 16) Results of DeSeq2 analysis showing significantly different abundances of 16S SVs between forests (protected versus fragmented) across species. The table reports the species, SV identifier, mean of normalized counts (baseMean), base 2 logarithm of the fold change (log2FoldChange), standard errors of the log2FoldChange (lfcSE), test statistics (stat), *P* values (pval), corrected *P* values (padj), and predicted taxonomy. (Sheet 17) Results of DeSeq2 analysis showing significantly different abundances of ITS SVs between forests (protected versus fragmented) across species. (Sheet 18) Mean and standard deviation (SD) of 16S SV importances estimated by the “habitat prediction” RF models trained on yellow baboons (*Papio cynocephalus*) and red colobus (*Procolobus gordonorum*) samples (Species column). (Sheet 19) Mean and standard deviation (SD) of ITS SV importances estimated by the “habitat prediction” RF models trained on yellow baboons (*Papio cynocephalus*) and red colobus (*Procolobus gordonorum*) samples (Species column). Download Data Set S1, XLSX file, 0.7 MB. Copyright © 2020 Barelli et al. 2020 Barelli et al. This content is distributed under the terms of the Creative Commons Attribution 4.0 International license.

The four most abundant phyla accounted for 84.6% and 75.3% of total bacterial SVs for baboons and red colobus, respectively, and the mean percentages of each differed between species. Due to the plausible implications of the *Firmicutes*/*Bacteroidetes* (F/B) ratio to obesity (46), this ratio has been suggested, despite limitations (47, 48), as an index of the health gut microbiome. Thus, we calculated F/B ratio between species and across habitats. The F/B ratio was significantly higher in yellow baboons than in red colobus (F/B ratio = 2.60 and 2.17, respectively; Wilcoxon rank sum test, *P* = 0.038), but within species, there was no difference between fragmented forest (FF) and protected forest (PF) (yellow baboons, *P* = 0.326; red colobus, *P* = 0.565; see Fig. S1 in the supplemental material).

10.1128/mSystems.00061-20.1 FIG S1 Violin plots indicating the distribution of the *Firmicutes*/*Bacteroidetes* ratio across species and forests. From the bottom to the top, the horizontal lines indicate the first, second (i.e., median), and third quartiles. Download FIG S1, PDF file, 0.03 MB. Copyright © 2020 Barelli et al. 2020 Barelli et al. This content is distributed under the terms of the Creative Commons Attribution 4.0 International license.

Among the 21 bacterial classes identified, 19 were detected in baboons, and 13 were detected in red colobus (Data Set S1, sheet 2), with *Clostridia* (mean relative abundances of 37% and 46.6% in baboons and red colobus, respectively; *P* = 1.11 × 10^−6^, Data Set S1, sheet 3) and *Bacteroidia* (10.3% and 19.8%, respectively; *P* = 7.06 × 10^−15^) being the two most abundant classes in both primate species. *Gammaproteobacteria* were highly represented in baboons (20.6% ± 19.9%), but not in red colobus (0.04% ± 0.2%; *P* = 5.39 × 10^−26^). At the family level, 36 microbial families were identified for baboons versus 21 for red colobus, with the two most abundant being the *Succinivibrionaceae* (20.2%) and the *Ruminococcaceae* (15.5%) in baboons and the *Lachnospiraceae* (22.1%) and *Ruminococcaceae* (16.8%) in red colobus (Data Set S1, sheet 4). The two primate species had very different distributions of unclassified bacteria: the fraction of SVs that could not be classified at the genus level reached a mean of 85.1% in red colobus samples but only 52.2% in yellow baboons (Fig. 1c).

Bacterial alpha diversity differed significantly across the two primate species. Both the Shannon index and number of observed SVs showed that red colobus have significantly higher diversity than yellow baboons (*P* = 2.3 × 10^−8^ and *P* = 7.2 × 10^−11^ for Shannon index and number of observed SVs, respectively; Wilcoxon rank sum test, Fig. 1b). Bray-Curtis dissimilarities also confirmed that bacterial compositions of the two host species were significantly different from each other, as is clear in Fig. 1d (permutational multivariate analysis of variance [PERMANOVA], *R*^2^= 0.296, *P* ≤ 0.0001).

**FIG 2:**
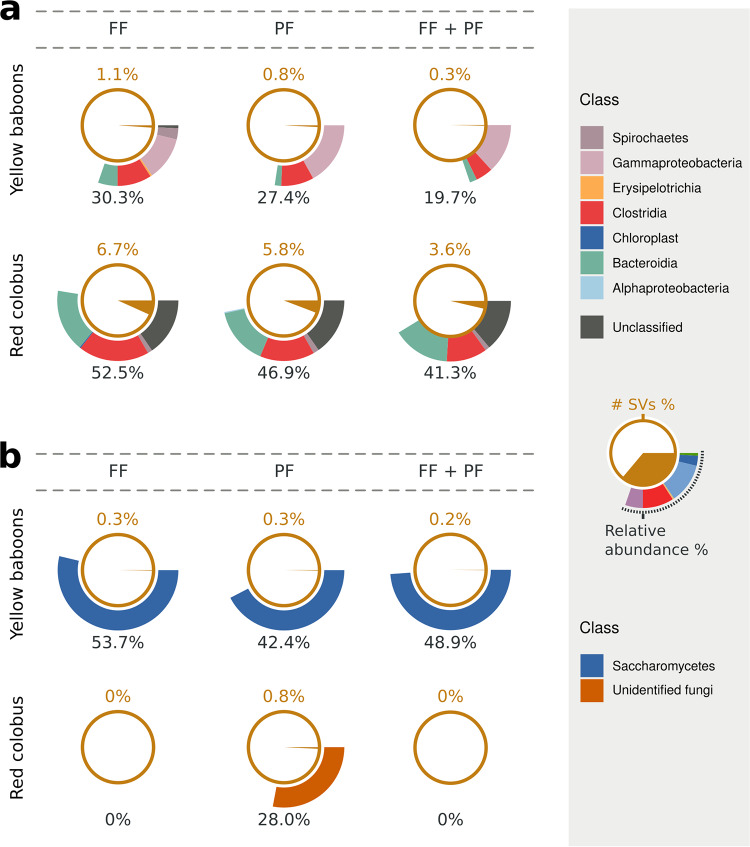
Core bacterial (a) and fungal (b) microbiota calculated for each forest separately (fragmented forest [FF] or protected forest [PF]) or combined (FF + PF) for yellow baboons (top pie charts) and red colobus (bottom pie charts). The core microbiota is expressed as the fraction of the number of SVs (inner brown pie charts and top brown numbers) and as mean relative abundance (separated at class level, outer rings and bottom black numbers).

Given a primate species and forest, a very low percentage of bacterial SVs were shared between individuals, even lower when results from the two habitats per species were combined (Fig. 2, inner pie charts). However, among the SVs present in at least 75% of the samples (bacterial “core communities”), those that were shared belonged to the classes representing 20 to 50% of the total relative abundances in each sample (except for red colobus fungal SVs; Fig. 2, outer rings). Yellow baboons shared only about 1% or less of SVs, in both single forests and when they were combined (Fig. 2a). Instead, red colobus shared about 3 to 7% in all combinations of habitats (Fig. 2a).

### (ii) Fungi: taxonomy, abundance, and diversity across hosts.

A total of 123 fungal families (79 in yellow baboons and 101 in red colobus) were identified (Data Set S1, sheet 5). These families derived primarily from three phyla: Ascomycota, Basidiomycota, and Zygomycota (Data Set S1, sheets 6 and 7). At the class level, significant differences between primate species were found in Sordariomycetes (*P* = 4.55 × 10^−8^), Saccharomycetes (*P* = 6.53 × 10^−22^), Dothideomycetes (*P* = 1.67 × 10^−3^), Agaricomycetes (*P* = 9.29 × 10^−4^), and Tremellomycetes (*P* = 1.15 × 10^−3^; Data Set S1, sheet 8). In contrast to bacterial SVs, the fraction of unclassified fungal SVs at the genus level was much lower, although higher in yellow baboons than in red colobus (23.7% and 12.3%, respectively; Fig. 1c and Data Set S1, sheet 9). Like bacteria, the fungal alpha diversity differed across host species with red colobus having significantly higher fungal richness than yellow baboons (Fig. 1b) regardless of the index (number of SVs, *P* = 1.1 × 10^−9^; Shannon entropy index, *P* = 6.7 × 10^−15^). Moreover, the internal transcribed spacer (ITS) profiles between species differed significantly (*R*^2^ = 0.182, *P* ≤ 10^−4^) in terms of Bray-Curtis dissimilarity (Fig. 1d).

As for bacteria, very few fungal SVs were shared between individuals of each species (Fig. 2). However, the core fungal composition of baboon fecal samples was dominated by a few SVs of the Saccharomycetes class that represented on average 50% of the relative abundances in both forests (Fig. 2b). Red colobus individuals shared virtually no fungal SVs, and those that they did share could not be identified at taxonomic levels lower than domain (Fig. 2b).

### (iii) Correlation between and within bacterial and fungal microbiota.

For each primate species, we also explored correlations between diversity indices between bacteria and fungi and within each domain. Only red colobus showed negatively correlated Shannon indices between domains (Pearson *r* = −0.301, *P* = 0.007; Fig. S2), but the same pattern was not noted in this species for other alpha diversity indices such as the number of observed SVs or Chao1. No correlation of alpha diversities between bacteria and fungi was detected in yellow baboons. *C*ompositionality *c*orrected by *r*enormalization and *pe*rmutation (CCREPE) analysis of relative abundances between domains in each species also indicated significant correlations between bacteria and fungi, mainly involving *Clostridia* and *Bacteroidia* from the bacterial classes and Sordariomycetes and Dothideomycetes among the fungal classes. Again, a greater number of correlations were found in red colobus (24 in Mwanihana; eight in Magombera; Data Set S1, sheet 10) compared to yellow baboons (only one correlation in Mwanihana; Data Set S1, sheet 11).

10.1128/mSystems.00061-20.2 FIG S2 MICTools analysis. Significant relationships between the relative abundances of SVs across species and habitats for both 16S (a) and ITS (b) data. SVs are grouped by the predicted taxonomy; the size of the circle represents the number of significant SV relationships for each pair of classes. Download FIG S2, PDF file, 0.01 MB. Copyright © 2020 Barelli et al. 2020 Barelli et al. This content is distributed under the terms of the Creative Commons Attribution 4.0 International license.

Considering relative abundances within each domain, we found significant positive and negative correlations in each primate species (MICtools analysis, see details in Data Set S1, sheets 12 to 15). Specifically, the numbers of correlations for fungi were similar between primate species, while many more correlations were noted for bacteria in baboons (Fig. S3). Again, the majority of correlations included unidentified fungi and *Clostridia* and *Bacteroidia* bacteria.

10.1128/mSystems.00061-20.3 FIG S3 Results of MICTools analysis, showing significant relationships between the relative abundances of SVs across species and habitats for both 16S (a) and ITS (b) data. SVs are grouped by predicted taxonomy; the size of the circle represents the number of significant SV relationships for each pair of classes. Download FIG S3, PDF file, 0.02 MB. Copyright © 2020 Barelli et al. 2020 Barelli et al. This content is distributed under the terms of the Creative Commons Attribution 4.0 International license.

### Are gut microbiota bacterial and fungal diversities higher in hosts inhabiting protected forests than in those living in fragmented and degraded ones? (i) Bacteria.

**FIG 3:**
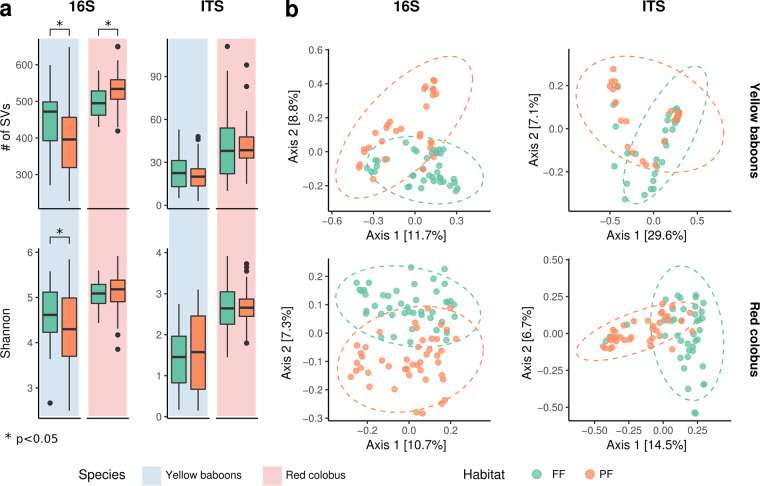
Diversity of bacterial and fungal gut communities in red colobus and yellow baboons in different forest types. (a) Alpha diversity of bacterial (16S) and fungal (ITS) communities estimated as number of observed SVs (top panel) and Shannon entropy (bottom panel) in yellow baboons (blue background) and red colobus (red background) in fragmented forest (FF) (green bars) and protected forest (PF) (orange bars). *, *P* < 0.05. (b) Principal coordinate analysis (PCoA) of Bray-Curtis dissimilarities of bacterial (16S) and fungal (ITS) data (see also Data Set S1, sheet 7, in the supplemental material) within species and across habitats. Dashed ellipses represent 95% confidence interval, while the numbers in square brackets refer to the variance explained by the PCoA axes.

Red colobus had a significantly higher number of observed bacterial SVs in PF than in FF (*P* = 0.036, generalized linear model), but not a higher Shannon entropy (*P* = 0.659; Fig. 3a). The opposite was found in yellow baboons, with individuals from FF having a significantly higher bacterial alpha diversity than those from PF for both indices (number of observed SVs and Shannon index: *P* = 0.002 and *P* = 0.003, respectively; Fig. 3a).

**FIG 4:**
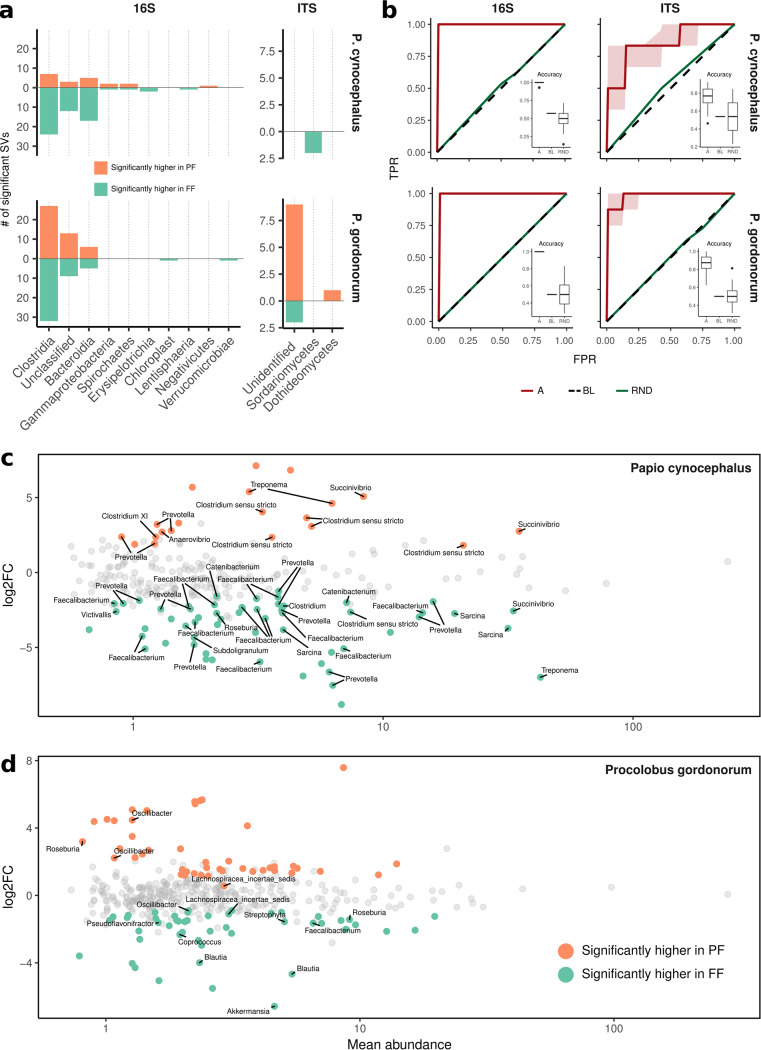
Significant differentially abundant SVs (*P* < 0.01) grouped by class (a) and genus (c) level. The green bars in panel a show SVs that are significantly higher in fragmented forest (FF), while the orange bars show SVs that are significantly higher in protected forest [PF]), estimated from the number of SVs. (b) Random Forest (RF) predictive performances (receiver operating characteristic [ROC] curves and accuracy [insets] are shown for both bacterial [left panel] and fungal [right panel] domains and across primate species [top and bottom plots]). Abbreviations: TPR, true positive rate; FPR, false-positive rate; A, actual; BL, baseline classifier; RND, random classifier. (c) Logarithm-transformed fold change (log2FC) of each SV against its mean relative abundance for yellow baboons (top panel) and red colobus (bottom panel). Significant SVs (colored points) without the taxonomic annotation are “Unclassified” SVs at the genus level.

*DESeq2* was used to identify changes in the relative abundance of taxa between primate species and habitat types. *DESeq2* identified differentially abundant bacterial SVs between forests, independently for each primate species (false discovery rate [FDR] corrected using the Benjamini-Hochberg procedure, *P* < 0.01; Fig. 4a, left panel), highlighting that among the 31 *Clostridia* SVs identified as differentially abundant in yellow baboons, 24 were more abundant in FF and 7 were more abundant in PF. Similarly, for 22 *Bacteroidia* differentially abundant in yellow baboons, 17 were more abundant in FF and five in PF. In addition, the classes *Erysipelotrichia* and *Lentisphaeria* were enriched only in FF and *Negativicutes* only in PF (Fig. 4a). For red colobus, 32 *Clostridia* SVs were differentially abundant in FF and 27 in PF; five *Bacteroidia* SVs were differentially abundant in FF and six in PF (Fig. 4a). *DESeq2* analyses also found that yellow baboon samples from FF were enriched in 10 genera; for example, 16 *Faecalibacterium* and 12 *Prevotella* (Fig. 4c), while the same species from PF was enriched with six other genera (Fig. 4c). Conversely, although most bacterial genera in red colobus were unclassified, nine were differentially abundant in FF and three in PF (Fig. 4c, bottom portion). Among those that could be classified, *Faecalibacterium*, *Coprococcus*, *Pseudoflavonifractor*, *Streptophyta*, *Blautia*, and *Akkermansia* were found only in FF (Fig. 4c, bottom portion; see also Data Set S1, sheet 16).

### (ii) Fungi.

Fungal diversity was not statistically different between habitats for either primate species (yellow baboons, *P* = 0.506, *P* = 0.395; red colobus, *P* = 0.741, *P* = 0.785; Fig. 3a).

Instead, comparison of beta diversity revealed that the composition of bacterial and fungal communities was unique for each species across habitats (Bray-Curtis dissimilarities and weighted UniFrac for 16S in yellow baboons, *R*^2^ = 0.067, *P* < 0.001 and *R*^2^ = 0.077, *P* < 0.001; in red colobus, *R*^2^ = 0.059, *P* < 0.001 and *R*^2^ = 0.042, *P* < 0.001; for ITS in yellow baboons, *R*^2^ = 0.036, *P* = 0.017 and *R*^2^ = 0.023, *P* = 0.17; in red colobus, *R*^2^ = 0.075, *P* = 0.001 and *R*^2^ = 0.080, *P* = 0.001; Fig. 3b; see also Table S1 in the supplemental material).

When considering the ratio between the number of 16S and ITS SVs, we did not find any significant differences (Wilcoxon rank sum test, *P* = 0.397) between individuals living in different habitats. However, yellow baboons had a higher ratio than red colobus did (*P* = 1.29 × 10^−4^; yellow baboons, median = 19.7, interquartile range [IQR] = 21.0; red colobus, median = 13.1, IQR = 8.94).

Fungal communities presented a smaller number of differentially abundant SVs than bacterial communities did (Fig. 4a, right panels): only yellow baboon samples from FF were enriched in two Sordariomycetes classes. Red colobus from FF had two differentially abundant unidentified classes, while those from PF were enriched in Dothideomycetes and nine unidentified classes (Fig. 4a; see also Data Set S1, sheet 17).

### Can microbiota composition be used to predict habitat origin?

Machine learning algorithms were used to test whether the origin of each sample from the composition of its bacterial and fungal microbiota can be predicted. The random forest classification algorithm revealed that sample origin could be predicted on the basis of bacterial composition for both red colobus and yellow baboons (for both species accuracy and Matthews correlation coefficient [MCC], median = 1, IQR = 0; out-of-bag [OOB] error [mean ± SD], yellow baboons, 0.03 ± 0.02; red colobus, 0.001 ± 0.004; Fig. 4b, left panels). The most relevant SVs explaining the habitat prediction model were distinct for red colobus and yellow baboons, with SVs belonging to the *Ruminococcaceae* and *Lachnospiraceae* families in red colobus and to the *Prevotellaceae* and *Clostridiaceae* in yellow baboons (Data Set S1, sheet 18; Fig. S4). Thus, the taxa involved in explaining the model were highly different between primate species, confirming the results found for the *DESeq2* analysis (Fig. 4c).

10.1128/mSystems.00061-20.4 FIG S4 Top 15 16S SVs in terms of feature importance identified by the habitat prediction RF models trained on yellow baboons (*Papio cynocephalus*) and red colobus (*Procolobus gordonorum*) samples. The thick colored bars and intervals represent, respectively, the means and 2 standard deviations of the normalized Gini importances calculated over the 50 cross-validation iterations. Download FIG S4, PDF file, 0.02 MB. Copyright © 2020 Barelli et al. 2020 Barelli et al. This content is distributed under the terms of the Creative Commons Attribution 4.0 International license.

Similarly, fungal composition also predicted sample origin (Fig. 4b, right panels) but with less accuracy (for yellow baboons, accuracy, median = 0.77, IQR = 0.15; MCC, median = 0.55, IQR = 0.32; for red colobus, accuracy, median = 0.88, IQR = 0.13; MCC, median = 0.76, IQR = 0.21; OOB error [mean ± SD] for yellow baboons, 0.28 ± 0.05; for red colobus, 0.14 ± 0.03). Although the most relevant SVs involved in explaining the model were “Unidentified” at the family level for red colobus, those SVs associated with habitat were again very different between species (Data Set S1, sheet 19; Fig. S5).

10.1128/mSystems.00061-20.5 FIG S5 Top 15 ITS SVs in terms of feature importance identified by the habitat prediction RF models trained on yellow baboons (*Papio cynocephalus*) and red colobus (*Procolobus gordonorum*) samples. The thick colored bars and intervals represent, respectively, the means and 2 standard deviations of the normalized Gini importances calculated over the 50 cross-validation iterations. Download FIG S5, PDF file, 0.02 MB. Copyright © 2020 Barelli et al. 2020 Barelli et al. This content is distributed under the terms of the Creative Commons Attribution 4.0 International license.

## DISCUSSION

Here, we test whether the bacterial and fungal components of the gut microbiota differ between two species of wild primates with different ecologies living in both protected and fragmented forests in a tropical African biodiversity hotspot. Because seasonal shifts may drive variation in dietary patterns and, consequently, in gut microbiota compositions (e.g., see references 21, 49, and 50), individual fecal sample collection was performed in a very narrow temporal window to avoid any potential seasonal effects. Thus, any intraspecific differences should reflect the effects of habitat type. Our findings revealed several patterns: bacterial and fungal microbiota composition of arboreal, leaf-eating red colobus monkeys was very different from that of ground-dwelling omnivorous yellow baboons, with red colobus having higher bacterial and fungal gut diversities than yellow baboons, as predicted from differences in dietary strategies. However, while red colobus living in protected forests had higher levels of diversity than those in unprotected forests, as predicted and noted in previous studies (4), unexpectedly, yellow baboons showed the opposite correlation, probably due to their ecologically different niches represented by their omnivorous diet and terrestrial habits. In addition, some correlations between bacterial and fungal diversity and composition were detected, and we showed that using machine learning models, it is possible to identify habitat of origin from fecal sample microbiota diversity. These results are discussed with regards to conservation issues and potential applications.

### Gut microbiota diversities are higher in the leaf-eating red colobus than in the omnivorous yellow baboons.

Despite the lack of explicit data on the diet ingested by the hosts in our results, we do refer to the extensive data on dietary habit and intake available from the literature of these and other red colobus and baboon populations. Bacterial richness (alpha diversity) was significantly higher in leaf-eating red colobus than in omnivorous yellow baboons, highlighting the differences in dietary strategies and digestive physiology of the two species. Although these two species live sympatrically and both belong to the *Cercopithecidae*, they possess ecologically different adaptations. Yellow baboons have an anatomically simple digestive tract, are hindgut fermenters and omnivorous, consuming a variety of food items, such as fruits, leaves, seeds, insects, roots, and small vertebrates (51) with 6% to 20% of fiber intake (52). Udzungwa red colobus, like other red colobus, have a specialized digestive system similar to ruminants, are foregut fermenters and rely almost exclusively on a folivorous diet, with up to 75% of fiber intake (53, 54). Although baboons occasionally include leaves and seeds in their diet, it is unlikely that their diet contains fiber contents comparable to that of red colobus. Thus, our results are in line with those revealing that greater fiber intake is associated with higher bacterial richness (41, 42, 55, 56). Similar findings have been reported for other primate species (20) and humans under strictly vegetarian diets compared to omnivorous ones (44). The two host species also showed highly different fungal richness, resembling the pattern found in bacteria but at a much lower scale (150-fold lower). Leaf-eating red colobus monkeys again presented higher fungal richness than the omnivorous baboons, consistent with previous findings (38) indicating that hosts with high gut fungal richness might be more efficient in breaking down plant fibers (57). Another interesting difference between the two host species concerns the surprisingly low interindividual variation of bacterial composition in red colobus compared to yellow baboons. Such stable and subtle interindividual variation likely indicates that red colobus gut bacteria are more phylogenetically similar because they have similar and highly specialized functions (i.e., degrading complex plant material such as cellulose, hemicellulose, and xylan [4]), compared to the gut bacteria of yellow baboons, which show greater dietary plasticity and greater ability to inhabit novel environments (i.e., human settings).

### Gut microbiota diversities are higher in protected forests for red colobus but not yellow baboons.

Ecological theory as well as empirical data have long shown that large expanses of natural forest habitat usually host a high autochthonous tree diversity, offering their wild animal inhabitants a more diverse diet than smaller tracts of degraded and fragmented habitat (58). Furthermore, it has been suggested that greater dietary variation promotes higher bacterial diversity in the host gut (59). Thus, if we consider the two host species living in intact and degraded habitats, we expected that higher bacterial alpha diversity should be found in the species residing in their natural, intact habitat. Our results strongly support this hypothesis for the red colobus, confirming our previous report noting associations between higher bacterial richness and habitat integrity (4), which are corroborated in other species as well (black howler monkey [*Alouatta pigra*] [5]; Andean bears [Tremarctos ornatus] [60]). However, baboons had an unexpectedly high gut bacterial diversity in FF compared to PF. This may be because fragmentation or disturbance of vegetation may stimulate a higher number of tolerant tree species in secondary-growth forest (61), and/or provoke modification in both diet composition and quality, allowing for a greater diversity of food consumption (62) in generalist feeders. Therefore, the higher bacterial diversity found in the omnivorous baboons could be due to a more diverse diet of baboons in these habitats. However, these differences in gut diversity could also be due to differences in behavior. Because of the rapid conversion of tropical forest to human-modified croplands, baboons have altered their socioecological behavior, especially in the neighboring areas where human density is the highest (like FF compared to PF), spending more time on the ground feeding on human food and food waste in villages, as well as raiding cultivations (i.e., vegetables and sugarcane). In this way, baboons increase their nutrient intake (63–65), but they also come into more frequent contact with soil, livestock, and humans, as well as their associated microbiota, either directly or through fecal-oral transmission. Higher gut flora diversity in mammals living on human-derived diets have been found in olive baboons [Papio anubis] (66), polar bears [Ursus maritimus] feeding on bone piles (67), and cattle straying into suburban areas (68), as well as in some species in captivity (including rhinoceros and red-crown crane [Grus japonensis] [69, 70] but see references 71 and 72). More recently, Grieneisen and colleagues have suggested that soil properties also alter soil microbiota and the microbiota of terrestrial animal species (73). Together, these results suggest that disturbed habitats may promote a higher diet diversity rather than a lower diet diversity and/or modify behavior, both of which contribute to the biodiversity of host gut communities. Since bacterial richness has been suggested as an indicator of “gut health,” either animals in proximity to human settlements and consuming human-derived food are healthier than those living in PF or microbiota richness is not in fact an indicator of gut health. For example, in the polar bear, the higher gut diversity of bears feeding on bone piles is also associated with a higher number of potential pathogens (67). Moreover, in light of new results (73), we cannot exclude the possibility that the soil microbiota of the two forests may directly shape microbial communities in this baboon population, regardless of diet and behavior.

### Gut microbiota composition and function.

A closer examination of more specific components of the bacterial and fungal microbiota confirms the relevance of the diverse dietary strategies of the two primate species in different habitats (41, 74). For example, leaf-eating red colobus monkeys shared, as expected, clades of the *Bacteroidia* and *Clostridia* classes most likely associated with the digestion of cellulose (75). In fact, the bacterial families *Ruminococcaceae* and *Lachnospiraceae* dominated the red colobus guts. Because those families are associated with degradation of complex plant material like cellulose and lignin (75, 76), the overrepresentation in red colobus may reflect their folivorous diet, as already found in most of the leaf-eating primates and ruminants (20, 77, 78). Our findings that baboon guts were predominantly associated with *Gammaproteobacteria* which are linked to starch digestion and carbohydrate fermentation confirm previous results (79, 80) and support their higher consumption of sugar-rich items. More specifically, baboon guts were dominated by *Succinivibrionaceae* (20.2%), which is a starch-digesting bacterial family that assists in the fermentation of carbohydrates to succinate and acetate (81). For fungi, the guts of yellow baboons were mainly dominated by Ascomycota (the largest phylum of the kingdom fungi), a few Basidiomycota and Zygomycota, consistent with previous findings in humans and nonhuman primates (30, 39, 82), whereas the guts of red colobus, beside considerable Ascomycota and a few Basidiomycota and Zygomycota, showed many unidentified fungi. An examination of core SVs follows this pattern: there were no shared bacterial and fungal SVs across species; however, within host species, a few core SVs accounted for about half of the microbiota composition.

Bacterial and fungal community composition also differed considerably across protected (PF) and fragmented (FF) habitats that just 60 years ago were naturally connected, before human settlements and intensive agriculture separated them (83). In fact, each species in each habitat had a unique gut microbiota, for both bacteria and fungi, again most likely linked to differences in diet discussed above. For example, among the classified SVs, only baboons of Magombera (FF), but not those of PF, were enriched with the bacterial genera *Sarcina*, *Prevotella*, and *Faecalibacterium. Sarcina* are synthesizers of microbial cellulose and linked to chitinolytic or protein-degrading functions, consistent with arthropod consumption, as reported for the white-faced capuchin monkeys (49). Since arthropods are a regular part of the diet of this and other baboons regardless of habitat (51, 84), the enrichment of *Sarcina* in the FF forest may be associated with feeding on a high-protein human-derived food resource, such as human waste. Indeed, *Prevotella* are frequently associated with consumption of fiber- and sugar-rich diets (40), consistent with the baboon diet in FF which includes sugarcane; similarly, *Prevotella* was also enriched in captive douc langurs consuming sugar-rich fruits (22). However, factors other than fiber and sugar ingestion contribute to high diversity and abundance; for example, *Sarcina* and *Prevotella* diversity are also linked with chronic gut inflammatory conditions and are implicated in causing gut diseases (85–87). Interestingly, in contrast to the potential negative associations of these genera, the butyrate-producing *Roseburia* and the *Faecalibacterium* genus were also overrepresented in baboons living in FF, two taxa generally associated with promoting intestinal barrier function and thus, metabolic benefits. On the other hand, baboons living in PF had a relatively high abundance of *Clostridium sensu stricto*, another group of bacteria providing protective mechanisms involved in the resistance to infection (88). More-detailed investigations on the role of such taxa are necessary to assess these interactions and gut homeostasis.

Considering the central role that fungi have in maintaining intestinal homeostasis and systemic immunity (89), variation in their diversity may impair host health and gut homeostasis. Thus, investigating either the direct interaction with the host and the competitive association between bacterial and fungal microorganisms in the gut become crucial for the conservation of animal species, especially for those that face constant threat due to habitat modification such as primate hosts. For fungi, we found that 10 different classes of unidentified fungi and one class of the Dothideomycetes were significantly enriched in PF in red colobus, presumably in response to a diet more rich in fiber. Baboons from FF were instead significantly enriched in two SVs belonging to the Sordariomycetes class. Although still poorly understood, the different dietary habits may drive diversification of fungal communities across species. However, given the lack of genomic databases and knowledge on the metabolic functions of fungi (compared to those of bacteria), at present, we can only note the differences and that patterns are similar to those found for bacteria, but we are unable to conjecture about potential explanations for these patterns.

The positive correlations between fungal and bacterial communities in the leaf-eating monkeys (especially those living in protected forests) and between bacterial taxa of baboons are intriguing, although their biological significance is still unclear, given the rarity of the taxa involved. Further investigation into these relationships are needed to unravel the significance of these patterns.

### The Udzungwa hotspot is associated with a high proportion of unclassified SVs.

About 10 to 20% of the total SVs could not be assigned even to a phylum as noted previously for bacteria (4). We contend that the high level of native vertebrate and invertebrate species reported thus far from the Udzungwa hotspot (90) is also reflected in primate gut microbiota, resulting in a high number of unclassifiable bacterial and fungal taxa unlisted in any database, and therefore, new to science, as also suggested for lemurs (91), Verreaux’s sifakas (50), and human hunter-gatherers (55). Interestingly, only about half of the yellow baboon gut bacteria are unidentified at the genus level compared to 85% of red colobus gut bacteria (Fig. 1c), possibly reflecting the higher contact between baboons and human residents, their omnivorous diet and similar digestive system, and therefore, possibly sources of gut microbiota (which is much better known for humans). However, further investigations of these unknown microrganisms are ongoing.

### Rethinking gut microbiota diversity as a tool for conservation.

Since greater taxonomic diversity might mirror greater functional diversity (9, 92), it is plausible that higher gut diversity might have an effect on “gut health” (93), and therefore, on overall host health (94, 95). Therefore, it has been suggested that gut microbiota could be used as a tool for assessing individual health of threatened taxa, especially in the case of noninvasive sampling (96). As mentioned above, the leaf-eating red colobus had higher bacterial and fungal richness than omnivorous baboons, indicating that their fiber-rich diet may promote a healthier gut; however, unexpectedly, baboons in FF had a higher gut diversity than baboons in PF. Thus, determining general patterns of associations between microbiota diversity and habitat characteristics remains elusive. However, we believe that species-specific ecological adaptations may mask the understanding of the general patterns (97). Based on gut microbiota diversity and composition, some species might be less resilient than others and respond differently to environmental variation. In this context, specialist leaf-eating primates, dependent on forest integrity, are more likely to show a greater impact to habitat degradation also in terms of gut microbiota diversity and composition. Conversely, species not dependent on a specialized diet, such as the omnivorous baboons that adapt better and show higher tolerance to human disturbance, may widen their dietary choices and potentially increase their gut microbiota. Although the possibility that omnivorous primates overcome the effects of habitat degradation by consuming human food and may be in better shape than those that are less unable to adapt like the red colobus is not excluded, we suggest that standard microbiota indices *per se* might not be a universal indicator of a “healthy gut.” Thus, deeper investigations into the functional analysis of each gut component are needed, as well as more appropriate data sets to better test ecological theory (98) for understanding which processes shape diversity in the host-associated microbial ecosystem. Moreover, to evaluate the association between gut health and diversity, and its value as a conservation tool, the present experimental design should be repeated using additional species, or even entire communities, with alternative lifestyles.

Besides the increasing impact of machine learning models on health care (99), to our knowledge, this is the first use of machine learning to investigate whether gut microbiota composition is associated with the habitat of origin in wildlife. Our analysis showed that almost all samples were correctly assigned to the forest where they were collected on the basis of their bacterial and fungal compositions. Therefore, the gut bacterial microbiota of each Udzungwa primate population is a distinctive signature of the forested area from which it belongs, and could potentially be used as a biomarker of their habitat of origin. Additionally, abiotic factors, such as soil properties and microbial communities of the surrounding environment, should be included to better predict host microbial diversity. Moreover, if gut microbiota accurately reflects the specificity of their habitat of origin, continued longitudinal monitoring of changes in this composition could indicate changes in habitat and/or individual health and species conservation status, as well as how quickly these are occurring. Further analyses of the data set to identify additional biomarkers for conservation and management purposes are also ongoing.

## MATERIALS AND METHODS

### Study site.

**FIG 5:**
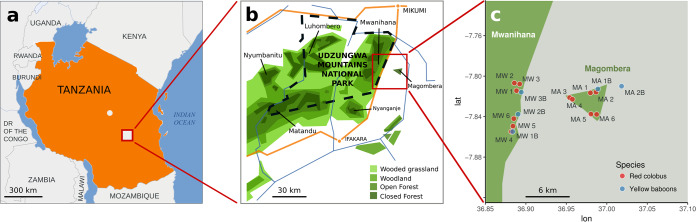
Map of the Udzungwa Mountains in Tanzania (a). Enlargement indicating the two forest blocks, Mwanihana and Magombera (b), and sample sites for the 12 social groups of Udzungwa red colobus and 5 social groups of yellow baboons (c). The dashed line in panel b indicates the border of the Udzungwa Mountain National Park. Colors identify species (i.e., red and blue for red colobus and yellow baboons, respectively).

The Udzungwa Mountains of Tanzania (Fig. 5) form part of an internationally recognized biodiversity hotspot (www.conservation.org); however, these relatively pristine forests are threatened by intensive agriculture (100), and primates living outside protected areas are particularly vulnerable to hunting and other human disturbance (101). The omnivorous yellow baboon is terrestrial, foraging for arthropods, seeds, fruit, and leaves, although they opportunistically also raid crops; this species is categorized as “least concern” by the International Union for the Conservation of Nature and Natural Resources (IUCN) (16). In contrast, the diet of the arboreal Udzungwa red colobus mainly consists of leaves and stems; it is native and endangered (16). Both these species live in Mwanihana (in a protected forest [PF]), an intact semideciduous to submontane and montane evergreen forest located within the boundaries of the Udzungwa Mountains National Park since 1992 (covering an area of 150 km^2^; altitude, 351 to 2,263 meters above sea level [ASL]), and Magombera (a fragmented forest [FF]) a flat, groundwater forest fragment surrounded by villages and sugarcane plantations approximately 6 km east of Mwanihana (covering an area of 12 km^2^; altitude, 269 to 302 meters ASL).

### Noninvasive fecal sample collection.

C. Barelli and two trained local field assistants followed the primate social groups (average group size, approximately 25 individuals) unobtrusively from the ground and collected fecal samples deposited by a total of 69 yellow baboons (belonging to five different social groups) and 89 red colobus (from 12 social groups; Fig. 5). The data collection was conducted over 4 weeks between June and July 2016 to avoid seasonal variation in diet (53) following already established procedures (4). Three social groups of yellow baboons were sampled from PF and two from FF. Six of the sampled groups of red colobus inhabited PF, while the remaining six groups were in FF. Samples were preserved in 96% ethanol, kept at 4°C, transported to the Fondazione E. Mach in Italy, and kept at –80°C until shipment to the University of Illinois, USA, for analysis. Sample collection complied with laws governing wildlife research in Tanzania under research permit 2016-267-ER-2009-49 obtained from the Tanzania Commission for Science and Technology (COSTECH), Tanzania Wildlife Research Institute (TAWIRI) and Tanzania National Parks (TANAPA).

### DNA extraction and Illumina sequencing.

Whole DNA was extracted from 0.25 g of each fecal sample using the QIAamp PowerFecal DNA kit (Qiagen Group, Hilden, Germany), following manufacturer’s instructions, quantified on a Qubit (Life Technologies) using the High Sensitivity DNA kit, and amplified by the DNA Services Laboratory, Roy J. Carver Biotechnology Center, University of Illinois, Urbana-Champaign, IL, USA. Library preparation for Illumina MiSeq sequencing of the V1-V3 regions of the 16S rRNA gene was carried out using the primer pair 28F and 519R for the PCR amplification of bacteria, while the 5′-GCATCGATGAAGAACGCAGC (forward) and 5′-TCCTCCGCTTATTGATATGC (reverse) primers were used to amplify the ITS1-ITS2 region of fungi (protocols found in references 121 and 122; further information in Text S1 in the supplemental material). Sterile water and human fecal DNA samples were also included as negative and positive controls during each step.

10.1128/mSystems.00061-20.7 TEXT S1 Detailed information of bacterial and fungal extraction methods, PCR amplification, and sequencing. Additional information on software used for data processing for both bacterial and fungal sequences and statistical analyses. Detailed scripts used to analyze bacterial (16S) and fungal (ITS) sequences for the Micca pipeline. Additional information on the intradomain relationships between SVs relative abundances, specifically, the correlations across classes of bacteria and fungi among the two primate hosts. Download Text S1, DOCX file, 0.02 MB. Copyright © 2020 Barelli et al. 2020 Barelli et al. This content is distributed under the terms of the Creative Commons Attribution 4.0 International license.

### Data processing and statistical analyses.

Raw bacterial 16S rRNA gene sequences were processed using the open-source MICCA (v1.7.0) software (102). After primer trimming, forward 16S reads shorter than 225 bp and with an expected error rate (103) of higher than 0.5% were discarded. Filtered sequences were denoised using the UNOISE (104) algorithm. Denoising methods (105–107) were chosen to correct sequencing errors and determine true biological sequences at the single nucleotide resolution by generating amplicon sequence variants (SVs) rather than operational taxonomic units (OTUs) defined using fixed similarity threshold (e.g., 97%) (108). Bacterial SVs were taxonomically classified using the Ribosomal Database Project (RDP) Classifier v2.11 (109). Multiple sequence alignments (MSA) were performed on the denoised reads applying the Nearest Alignment Space Termination (110) (NAST) algorithm, and the phylogenetic tree was inferred using FastTree v2.1.8 (110, 111).

Raw overlapping internal transcribed spacer (ITS) paired-end reads were assembled using the procedure described in reference 103. Paired-end reads with an overlap length smaller than 50 and with more than 15 mismatches were discarded. After forward and reverse primer trimming, merged reads shorter than 225 bp and with an expected error rate higher than 0.5% were removed. Filtered sequences were denoised as described above, and SVs were classified using the RDP Classifier v2.11 and the UNITE (112) database. Multiple sequence alignment was performed on SVs using MUSCLE v3.8.31 (113), and a phylogenetic tree was inferred using FastTree. Finally, SVs with less than 75% similarity to the sequences present in the UNITE database (clustered at 85%; release 2017/12/01) were discarded using VSEARCH v2.3.4 (114). The complete list of commands is reported in Text S1 in the supplemental material.

Downstream analyses were performed using R v3.4.2 with the *phyloseq* v1.25.3 and *vegan* v2.5-2 packages (115). 16S and ITS data from 158 individuals were rarefied (without replacement) at 2,400 reads per sample resulting in 16 ITS samples being discarded, as well as two 16S and 49 ITS SVs. Wilcoxon rank sum tests were performed to detect significant differences in relative abundances between the red colobus and the yellow baboon samples at the phylum, class, and family levels. *P* values were corrected using the Benjamini-Hochberg procedure. For each taxon, we estimated the median (and the 95% nonparametric confidence interval) of the relative abundance difference between the two species. Alpha diversity indices (observed number of SVs and Shannon entropy) for each fecal sample were estimated using the *phyloseq* library. The comparison of alpha diversity indices between the primate species was performed using the Wilcoxon rank sum test implemented in the R package *stats*.

To determine whether the core microbiota of each primate species was influenced by habitat, the number of SVs shared across individuals within each species was estimated overall and for each forest type. Core taxa were established by considering SVs present at least one time (on the rarefied SV matrix) and in at least 75% of samples, and between-forest richness analysis was performed using generalized linear models (GLM) available in the R package *stats*, with quasi-Poisson (*log* link function) and Gaussian families for the number of observed SVs and Shannon entropy, respectively. Permutational multivariate analyses of variance were performed by the function *adonis* (available in the R package *vegan*) on Bray-Curtis dissimilarities and weighted UniFrac distances (with 9,999 permutations). Differential abundance testing was carried out removing the 16 ITS samples with less than 2,400 reads and using the R package *DESeq2* (116) using the nonrarefied data as suggested in reference 117. Only SVs with at least five counts in more than 10 samples were considered in this analysis. *P* values were false discovery rate corrected using the Benjamini-Hochberg procedure implemented in *DESeq2*.

To infer whether bacterial and fungal profiles could predict the habitat of sample origin, random forest predictive classification models were built using the Python package *scikit-learn* (118). Generalization accuracy, Matthews correlation coefficient (MCC), out-of-bag (OOB) errors, and receiver operating characteristic (ROC) curves were estimated by a stratified random subsampling cross-validation (CV) scheme (50 splits) using the rarefied data. For each cross-validation split, the model was fitted using 90% of samples, and the classification accuracy was computed predicting the remaining 10% (blind set). Models were trained on rarefied SVs in order to avoid biases due to different sequencing depths. The number of estimators in the RF classifier was set at 500, and no model selection was performed. The RF feature importances were computed as the average of the normalized Gini importances calculated in each decision tree. Baseline accuracy values were calculated by a “dummy” classifier, which predicts the most frequent label in the training set. The “random” accuracy values were calculated generating random predictions according to the class distribution of the training set.

By combining available bacterial and fungal sequencing data from the same fecal samples, intradomain correlation analyses were performed using MICtools (119). All comparisons of relationships (i.e., bacterium-bacterium, fungus-fungus, fungus-bacterium within host species and forest) were assessed based on the relative abundance of bacterial and fungal SVs. Only SVs with a relative abundance higher than 0.1% in at least 30 samples were included in this analysis. To detect significant interactions between bacteria and fungi SVs, we additionally use the CCREPE R library (http://huttenhower.sph.harvard.edu/ccrepe) version 1.20.0. The number of bootstrap and permutation iterations was set at 100, and *P* values were FDR corrected using the *qvalue* (120) R package.

### Data availability.

DNA sequences have been deposited in the European Nucleotide Archive (ENA) under study accession number PRJEB37770. 16S and ITS raw sequences generated for this study and metadata are publicly available at https://doi.org/10.5281/zenodo.3725526. The machine learning algorithm is available at the repository link: https://github.com/compmetagen/barelli_et_al_msystems_2020.

10.1128/mSystems.00061-20.6 TABLE S1 Permutational multivariate analysis of variance (PERMANOVA) independently applied for each marker (16S and ITS) and species, using Bray-Curtis dissimilarity and weighted UniFrac distance. The table reports the F statistics (F), partial *R*-squared (*R*^2^), and uncorrected *P* values [Pr(>F)]. Download Table S1, DOCX file, 0.01 MB. Copyright © 2020 Barelli et al. 2020 Barelli et al. This content is distributed under the terms of the Creative Commons Attribution 4.0 International license.
