# Supplementary figures and images for: The Gut Microbiota Communities of Wild Arboreal and Ground-Feeding Tropical Primates Are Affected Differently by Habitat Disturbance
Source: mSystems. 2020 May 26;5(3):e00061-20. doi: 10.1128/mSystems.00061-20 (PMC7253362; doi:10.1128/mSystems.00061-20)

Firmicutes-Bacteroidetes ratio

Habitat

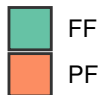

Yellow baboons

Red colobus

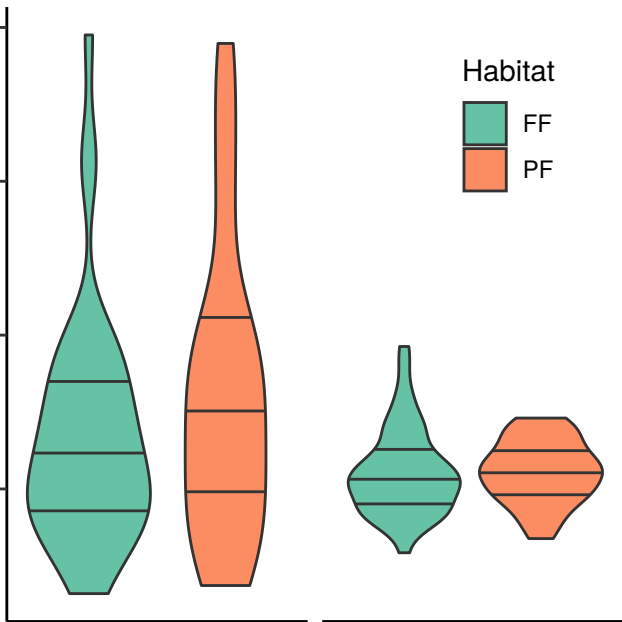

Supplement: FIG S1 [file mSystems.00061-20-sf001.pdf]

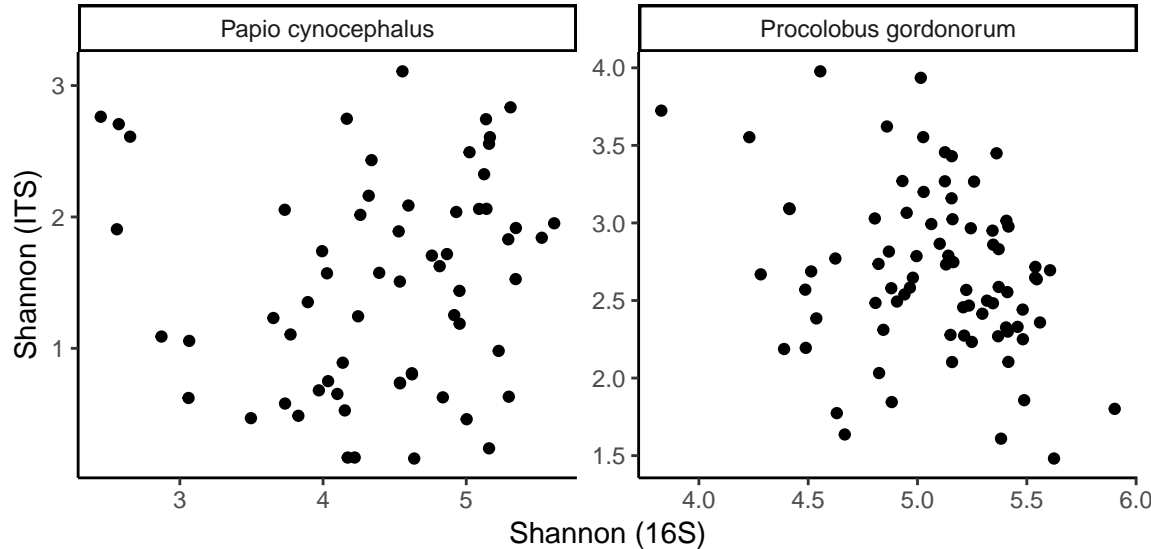

Supplement: FIG S2 [file mSystems.00061-20-sf002.pdf]

**a**

# of significant relationships

• 1

● 5

● 10

● 15

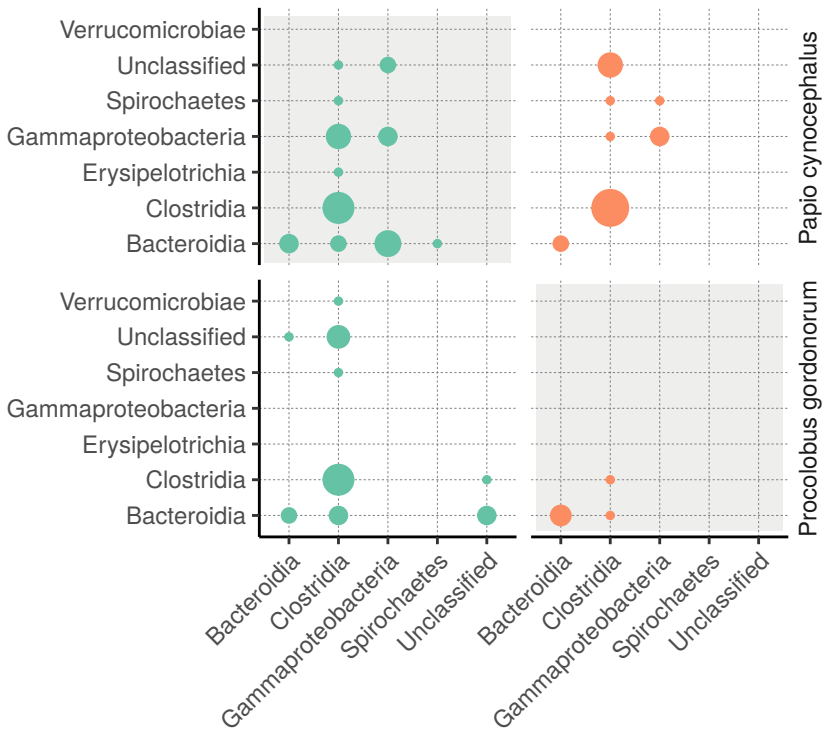**b**

# of significant relationships

• 1

● 5

● 10

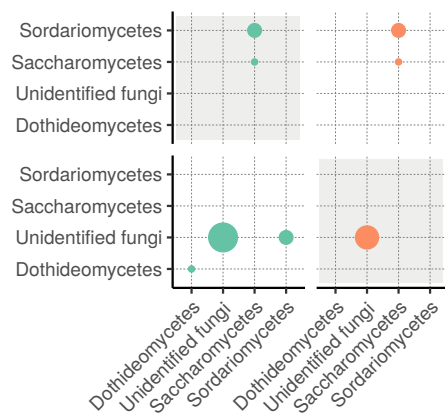

Supplement: FIG S3 [file mSystems.00061-20-sf003.pdf]

SV

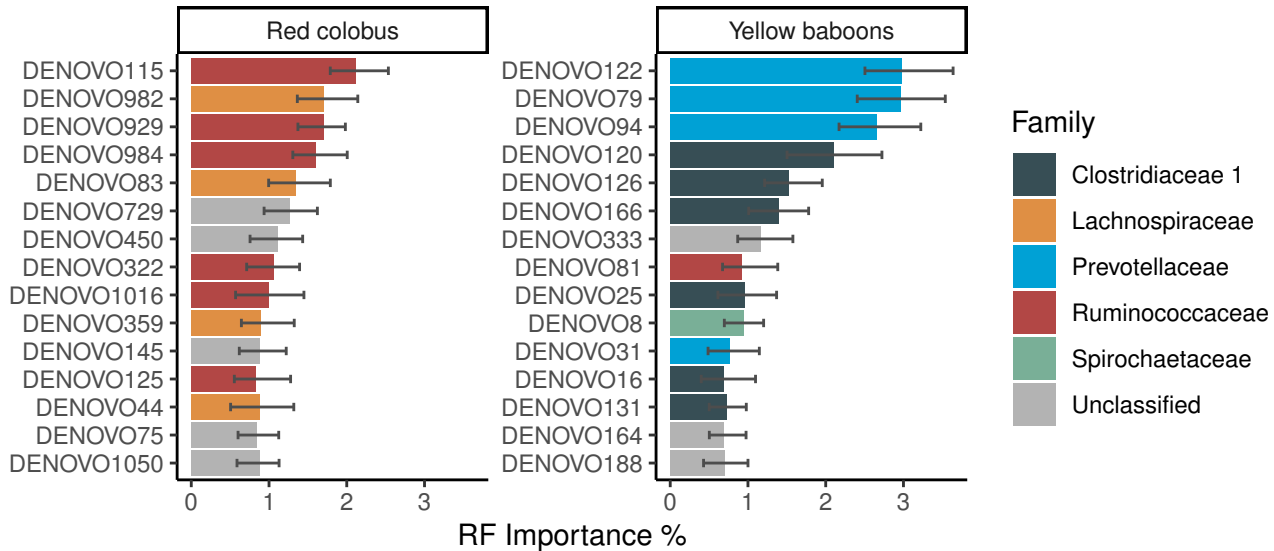

Supplement: FIG S4 [file mSystems.00061-20-sf004.pdf]

SV

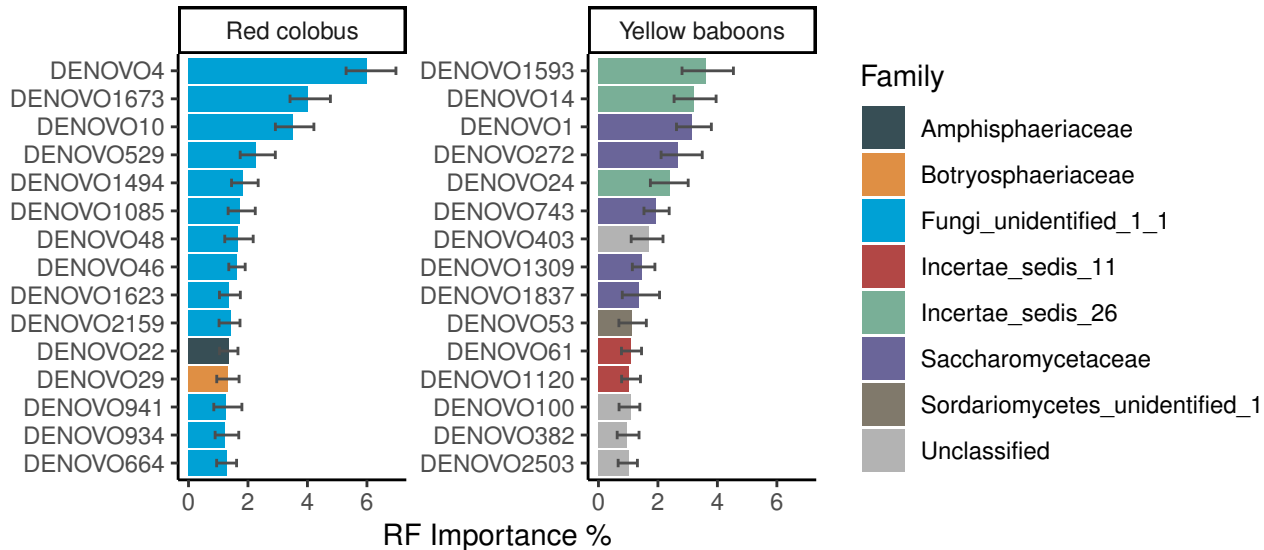

Supplement: FIG S5 [file mSystems.00061-20-sf005.pdf]
