# Supplementary material for: The Gut Microbiota Communities of Wild Arboreal and Ground-Feeding Tropical Primates Are Affected Differently by Habitat Disturbance
Source: mSystems. 2020 May 26;5(3):e00061-20. doi: 10.1128/mSystems.00061-20 (PMC7253362; doi:10.1128/mSystems.00061-20)
Supplement: TEXT S1 [file mSystems.00061-20-s0001.docx]

## **The gut microbiota communities of wild arboreal and ground-feeding tropical primates are affected differently by habitat disturbance**

###

### **Supplementary Information**

# Claudia Barelli^1,2,3^, Davide Albanese^4^, Rebecca M. Stumpf^3,5^, Abigail Asangba^5^, Claudio Donati^4^, Francesco Rovero^2,6^ & Heidi C. Hauffe^1^

^1^ Department of Biodiversity and Molecular Ecology, Research and Innovation Centre, Fondazione Edmund Mach, Via E. Mach 1, 38010 S. Michele all’Adige, Italy

^2^ Tropical Biodiversity Section, MUSE – Museo delle Scienze, Corso del Lavoro e della Scienza 3, 38123 Trento, Italy

^3^ Carl R. Woese Institute for Genomic Biology, University of Illinois, 1206 W Gregory Dr, 61801 Urbana, IL, USA

^4^ Unit of Computational Biology, Research and Innovation Centre, Fondazione Edmund Mach, Via E. Mach 1, 38010 S. Michele all’Adige, Italy

^5^ Department of Anthropology, University of Illinois, 607 S Mathews Avenue, 61801 Urbana, IL, USA

^6^ Department of Biology, University of Florence, Via Madonna del Piano 6, 50019 Sesto Fiorentino, Italy

**Supplementary Methods**

**DNA extraction and PCR amplification**

Bacterial and fungal communities were characterized from 0.25g of each fecal sample. DNA was extracted using the QIAamp PowerFecal DNA Kit (QIAGEN Group, Hilden, Germany), following manufacturer's instructions, including the bead-beating procedure. Whole DNA was amplified by the DNA Services Laboratory at the Roy J. Carver Biotechnology Center at the University of Illinois, Urbana-Champaign, IL, USA. Prior to amplification, DNA was quantified on a Qubit (Life Technologies) using the High Sensitivity DNA Kit. Samples were diluted to 2 ng/ul and a mastermix for amplification was prepared using the Roche High Fidelity Fast Start Kit and 20x Access Array loading reagent according to Fluidigm protocols. Final primer concentration in the reactions was 50 nM each. Primer sequences for amplification of the V1-V3 regions of the 16S ribosomal RNA gene were 28F 5'-GAGTTTGATCNTGGCTCAG (forward primer) and 519R 5'-GTNTTACNGCGGCKGCTG (reverse primer), and for the ITS1-ITS2 region, 5'-GCATCGATGAAGAACGCAGC (forward) and 5'-TCCTCCGCTTATTGATATGC (reverse). All primers were synthesized by IDT Corp. (Coralville, IA) and placed in the Juno microfluidic machine (Fluidigm Corp.) for loading all primer/sample combinations, followed by amplification and harvesting. Harvested products were randomly transferred to a new 96 well plate, quantified on a Qubit fluorimeter and stored at -20°C. All samples were run on a Fragment Analyzer (Advanced Analytics, Ames, IA) and amplicon regions and expected sizes confirmed. Samples were then pooled in equal amounts according to product concentration, size selected on a 2% agarose E-gel (Life Technologies), and extracted from the isolated gel slice with Qiagen gel extraction kit (Qiagen) using a Qiacube robot. Cleaned size selected products were run on an Agilent Bioanalyzer to confirm appropriate profile and determination of average size. The final pool was denatured and spiked with 20% non-indexed PhiX control library provided by Illumina and loaded onto the MiSeq v2 (500 cycle) flowcell at a concentration of 8 pM for cluster formation and sequencing. The PhiX control library provides a balanced genome for calculation of matrix, phasing and pre-phasing, which are essential for accurate base calling. The libraries were sequenced from both ends of the molecules to a total read length of 250 nt from each end. Water and human samples were also included for both bacterial and fungal extractions to act as a negative and positive controls during sequencing.

**Data processing and statistical analyses**

Raw bacterial 16S rRNA gene sequences were processed using the open-source MICCA (v1.7.0) software [[1]](https://paperpile.com/c/jR35R7/K16w) see Supplementary Material for details). After primer trimming, forward 16S reads shorter than 225 bp and with an expected error rate [[2]](https://paperpile.com/c/jR35R7/R1Cc) higher than 0.5% were discarded. Filtered sequences were denoised using the UNOISE [[3]](https://paperpile.com/c/jR35R7/WJB8) algorithm. Denoising methods [[4–6]](https://paperpile.com/c/jR35R7/GG6O+xDbK+zV7S) were chosen to correct sequencing errors and determine true biological sequences at the single nucleotide resolution by generating amplicon sequence variants (SVs) rather than OTUs defined using fixed similarity threshold (e.g. 97%) [[7]](https://paperpile.com/c/jR35R7/foKu). Bacterial SVs were taxonomically classified using the Ribosomal Database Project (RDP) Classifier v2.11 [[8]](https://paperpile.com/c/jR35R7/bUIm). Multiple sequence alignments (MSA) were performed on the denoised reads applying the Nearest Alignment Space Termination [[9]](https://paperpile.com/c/jR35R7/whd8) (NAST) algorithm and the phylogenetic tree was inferred using FastTree v2.1.8 [[10, 11]](https://paperpile.com/c/jR35R7/1h89+h393).

Raw overlapping ITS paired-end reads were assembled using the procedure described in [[12]](https://paperpile.com/c/jR35R7/mRie). Paired-end reads with an overlap length smaller than 50 and with more than 15 mismatches were discarded. After forward and reverse primer trimming, merged reads shorter than 225 bp and with an expected error rate higher than 0.5% were removed. Filtered sequences were denoised as above and SVs were classified using the RDP Classifier v2.11 and the UNITE [[13]](https://paperpile.com/c/jR35R7/wURf) database. Multiple sequence alignment was performed on SVs using MUSCLE v3.8.31 [[14]](https://paperpile.com/c/jR35R7/FbcI) and a phylogenetic tree was inferred using FastTree. Finally, SVs with less than 75% similarity to the sequences present in the UNITE database (clustered at 85%, release 2017/12/01) were discarded using VSEARCH v2.3.4 [[15]](https://paperpile.com/c/jR35R7/yQ10). The complete list of commands is reported in the Supplementary Material.

**Micca pipeline (16S)**

micca merge -i *_R1.fastq -o merged.fastq -s "_R1" micca trim -i merged.fastq -o trimmed.fastq -w GAGTTTGATCNTGGCTCAG –W micca filter -i trimmed.fastq -o filtered.fasta -e 0.50 -m 225

micca otu -m denovo_unoise -i filtered.fasta -o denovo_unoise_otus –c micca classify -m rdp -i denovo_unoise_otus/otus.fasta -o \ denovo_unoise_otus/taxa.txt --rdp-minconf 0.65 micca msa -m nast -i denovo_unoise_otus/otus.fasta -o \

denovo_unoise_otus/msa.fasta --nast-template core_set_aligned.fasta.imputed micca tree -i denovo_unoise_otus/msa.fasta -o denovo_unoise_otus/tree.tree micca root -i denovo_unoise_otus/tree.tree -o denovo_unoise_otus/tree_rooted.tree

**Micca pipeline (ITS)**

micca mergepairs -i *_R1.fastq -o merged.fastq --notmerged-fwd \

notmerged_fwd.fastq --notmerged-rev notmerged_rev.fastq -l 50 -d 15 -s "_R1" micca trim -i merged.fastq -o trimmed.fastq -w GCATCGATGAAGAACGCAGC \ -r TCCTCCGCTTATTGATATGC -W -R –c micca filter -i trimmed.fastq -o filtered.fasta -e 0.50 -m 225 micca otu -m denovo_unoise -i filtered.fasta -o denovo_unoise_otus micca classify -m rdp --rdp-gene fungalits_unite -i denovo_unoise_otus/otus.fasta \

-o denovo_unoise_otus/taxa.txt --rdp-minconf 0.65 micca msa -i denovo_unoise_otus/otus.fasta -o denovo_unoise_otus/msa.fasta \ --muscle-maxiters 4 micca tree -i denovo_unoise_otus/msa.fasta -o denovo_unoise_otus/tree.tree micca root -i denovo_unoise_otus/tree.tree -o denovo_unoise_otus/tree_rooted.tree vsearch --usearch_global denovo_unoise_otus/otus.fasta --db \ sh_refs_qiime_ver7_99_to_85_vsearch.fasta --id 0.75 --strand both --query_cov \ 0.75 --matched denovo_unoise_otus/otus_matched.fasta --notmatched \ denovo_unoise_otus/otus_notmatched.fasta

**Intra-domain relationships between SVs relative abundances**

We identified a greater number of significant bacterial correlations among yellow baboons (12 pairs of relationships between bacterial classes) compared to red colobus (9 relationships). Regardless of host species, most of the FF individuals presented a greater number of interactions than those living in PF. Red colobus compared to yellow baboons showed few interactions in terms of actual number and number of significant relationships (Fig. S3a). Despite some bacterial correlations only occurring in one habitat type, the number of significant relationships also differed. For example, among the classes which could be classified, only red colobus living in FF presented one significant correlation between Spirochetes/Clostridia, and one between Verrucomicrobiae/Clostridia. Similarly, only yellow baboons living in FF had 2 significant correlations between Bacteroidia/Clostridia, 7 between Bacteroidia/Gammaproteobacteria and 1 between Bacteroidia/Spirochetes (Fig. S3a).

Fungal correlations based on their relative abundance were clearly less evident (Fig. S3b). However, while yellow baboons living in both types of forest presented the same number of correlations between fungal classes, red colobus living in FF possess a greater number of them (8 vs 3 relationships of fungal classes; Fig. S3b). Moreover, the number of significant correlations in FF were much higher rather than in PF (16 vs 9).

**REFERENCES (Supplementary Methods)**

1. [Albanese D, Fontana P, De Filippo C, Cavalieri D, Donati C. MICCA: a complete and accurate software for taxonomic profiling of metagenomic data. *Sci Rep* 2015; **5**: 9743.](http://paperpile.com/b/jR35R7/K16w)

2. [Edgar RC, Flyvbjerg H. Error filtering, pair assembly and error correction for next-generation sequencing reads. *Bioinformatics* 2015; **31**: 3476–3482.](http://paperpile.com/b/jR35R7/R1Cc)

3. [Edgar RC. UNOISE2: improved error-correction for Illumina 16S and ITS amplicon sequencing. 2016.](http://paperpile.com/b/jR35R7/WJB8)

4. [Amir A, McDonald D, Navas-Molina JA, Kopylova E, Morton JT, Zech Xu Z, et al. Deblur Rapidly Resolves Single-Nucleotide Community Sequence Patterns. *mSystems* 2017; **2**.](http://paperpile.com/b/jR35R7/GG6O)

5. [Callahan BJ, McMurdie PJ, Rosen MJ, Han AW, Johnson AJA, Holmes SP. DADA2: High-resolution sample inference from Illumina amplicon data. *Nat Methods* 2016; **13**: 581–583.](http://paperpile.com/b/jR35R7/xDbK)

6. [Nearing JT, Douglas GM, Comeau AM, Langille MGI. Denoising the Denoisers: an independent evaluation of microbiome sequence error-correction approaches. *PeerJ* 2018; **6**: e5364.](http://paperpile.com/b/jR35R7/zV7S)

7. [Callahan BJ, McMurdie PJ, Holmes SP. Exact sequence variants should replace operational taxonomic units in marker-gene data analysis. *ISME J* 2017; **11**: 2639–2643.](http://paperpile.com/b/jR35R7/foKu)

8. [Wang Q, Garrity GM, Tiedje JM, Cole JR. Naive Bayesian classifier for rapid assignment of rRNA sequences into the new bacterial taxonomy. *Appl Environ Microbiol* 2007; **73**: 5261–5267.](http://paperpile.com/b/jR35R7/bUIm)

9. [DeSantis TZ, Hugenholtz P, Keller K, Brodie EL, Larsen N, Piceno YM, et al. NAST: a multiple sequence alignment server for comparative analysis of 16S rRNA genes. *Nucleic Acids Res* 2006; **34**: W394–W399.](http://paperpile.com/b/jR35R7/whd8)

10. [DeSantis TZ, Hugenholtz P, Keller K, Brodie EL, Larsen N, Piceno YM, et al. NAST: a multiple sequence alignment server for comparative analysis of 16S rRNA genes. *Nucleic Acids Res* 2006; **34**: W394–W399.](http://paperpile.com/b/jR35R7/1h89)

11. [Price MN, Dehal PS, Arkin AP. FastTree 2 – Approximately Maximum-Likelihood Trees for Large Alignments. *PLoS One* 2010; **5**: e9490.](http://paperpile.com/b/jR35R7/h393)

12. [Edgar RC, Flyvbjerg H. Error filtering, pair assembly and error correction for next-generation sequencing reads. *Bioinformatics* 2015; **31**: 3476–3482.](http://paperpile.com/b/jR35R7/mRie)

13. [Kõljalg U, Larsson K-H, Abarenkov K, Nilsson RH, Alexander IJ, Eberhardt U, et al. UNITE: a database providing web-based methods for the molecular identification of ectomycorrhizal fungi. *New Phytol* 2005; **166**: 1063–1068.](http://paperpile.com/b/jR35R7/wURf)

14. [Edgar RC. MUSCLE: multiple sequence alignment with high accuracy and high throughput. *Nucleic Acids Res* 2004; **32**: 1792–1797.](http://paperpile.com/b/jR35R7/FbcI)

15. [Rognes T, Flouri T, Nichols B, Quince C, Mahé F. VSEARCH: a versatile open source tool for metagenomics. *PeerJ* 2016; **4**: e2584.](http://paperpile.com/b/jR35R7/yQ10)
