# Supplementary material for: The Gut Microbiota Communities of Wild Arboreal and Ground-Feeding Tropical Primates Are Affected Differently by Habitat Disturbance
Source: mSystems. 2020 May 26;5(3):e00061-20. doi: 10.1128/mSystems.00061-20 (PMC7253362; doi:10.1128/mSystems.00061-20)
Supplement: TABLE S1 [file mSystems.00061-20-st001.docx]

**Supplementary Table S1**

| **Dissimilarity/Distance** | **Marker** | **Species** | **F** | **R^2^** | **Pr(>F)** |
| --- | --- | --- | --- | --- | --- |
| Bray-Curtis | 16S | *Papio cynocephalus* | 4.8279 | 0.06722 | 1.00E-04 |
|  |  | *Procolobus gordonorum* | 5.4844 | 0.0593 | 1.00E-04 |
|  | ITS | *Papio cynocephalus* | 2.3112 | 0.0365 | 0.0174 |
|  |  | *Procolobus gordonorum* | 6.2837 | 0.07545 | 1.00E-04 |
| Weighted UniFrac | 16S | *Papio cynocephalus* | 5.6179 | 0.07736 | 3.00E-04 |
|  |  | *Procolobus gordonorum* | 3.7868 | 0.04171 | 0.0012 |
|  | ITS | *Papio cynocephalus* | 1.4152 | 0.02267 | 0.1746 |
|  |  | *Procolobus gordonorum* | 6.6682 | 0.0797 | 1.00E-04 |
